# Upadacitinib improves patient-reported outcomes in patients with rheumatoid arthritis and inadequate response to conventional synthetic disease-modifying antirheumatic drugs: results from SELECT-NEXT

**DOI:** 10.1186/s13075-019-2037-1

**Published:** 2019-12-09

**Authors:** Vibeke Strand, Janet Pope, Namita Tundia, Alan Friedman, Heidi S. Camp, Aileen Pangan, Arijit Ganguli, Mahesh Fuldeore, Debbie Goldschmidt, Michael Schiff

**Affiliations:** 1 Stanford University, Palo Alto, CA USA; 2 University of Western Ontario, London, ON Canada; 3 AbbVie Inc., North Chicago, IL USA; 4 Analysis Group, Inc., New York, NY USA; 5 University of Colorado, Denver, CO USA

**Keywords:** Rheumatoid arthritis, Patient-reported outcomes, JAK inhibitor, Health-related quality of life, Treatment outcomes

## Abstract

**Background:**

To evaluate the effect of upadacitinib on patient-reported outcomes (PROs) in patients with RA who had an inadequate response to csDMARDs.

**Methods:**

Patients in SELECT-NEXT, a randomised controlled trial, were on a background of csDMARDs and received upadacitinib 15 mg and 30 mg or placebo daily for 12 weeks. PROs included Patient Global Assessment of Disease Activity (PtGA), pain, Health Assessment Questionnaire-Disability Index (HAQ-DI), Functional Assessment of Chronic Illness Therapy-Fatigue (FACIT-F), duration and severity of morning (AM) joint stiffness, Short Form 36 Health Survey (SF-36), and Work Instability Scale for RA (RA-WIS). Least squares mean (LSM) changes were based on mixed-effect repeated measure models. Percentages of patients reporting improvements ≥ minimum clinically important differences (MCIDs) and scores ≥ normative values and number needed to treat (NNT) were determined; group comparisons used chi-square tests.

**Results:**

Data from 661 patients were analysed. Compared with placebo, patients receiving upadacitinib reported statistically significant improvements (both doses, *P* < 0.05) in PtGA, pain, HAQ-DI, FACIT-F, duration and severity of AM stiffness, SF-36 (PCS and 6/8 domains), and RA-WIS at week 12. Significantly, more upadacitinib-treated patients (both doses, *P* < 0.05) reported improvements ≥ MCID in PtGA, pain, HAQ-DI, FACIT-F, AM stiffness, SF-36 (PCS and 4 or 7/8 domains), and RA-WIS and scores ≥ normative values in HAQ-DI, FACIT-F, and SF-36 (PCS and 4 or 5/8 domains). For most PROs, the incremental NNT with upadacitinib to report clinically meaningful improvement from baseline ranged from 4 to 8 patients.

**Conclusions:**

Upadacitinib 15 mg or 30 mg daily for 12 weeks resulted in significant and clinically meaningful improvements in global disease activity, pain, physical function, fatigue, duration and severity of AM stiffness, HRQOL, and work instability among csDMARD-IR patients with RA.

**Trial registration:**

Clinicaltrials.gov, NCT02675426. Retrospectively registered 5 February 2016.

## Background

Rheumatoid arthritis (RA) is a chronic, inflammatory, and destructive joint disease that is associated with substantial clinical burden [1]. Pain, fatigue, and morning (AM) stiffness are common symptoms associated with RA [2–6] and have an important negative impact on health-related quality of life (HRQOL) [7–9] and ability to work [10–12]. A recent survey identified pain, fatigue, and independence as the most important domains of RA disease activity that need to improve in patient-perceived remission [13]. Core patient-reported outcomes (PROs) including global assessment of disease, pain, physical function, fatigue, HRQOL, and work stability provide valuable insights into patients’ perspectives on their health status and impact of disease—improvements in PROs are considered important when evaluating the benefits of treatments [14–18]. Capturing the patient experience with these outcomes provides important information that can be used by clinicians to guide treatment decisions [19].

Janus kinase (JAK) inhibitors are a class of orally administered targeted synthetic disease-modifying antirheumatic drugs (tsDMARDs) that have recently received regulatory approval and are under evaluation in randomised controlled trials (RCTs) for the treatment of RA [20–23]. Upadacitinib, a selective JAK1 inhibitor, has demonstrated efficacy and a favourable benefit-to-risk profile in active RA among patients with inadequate responses to conventional synthetic disease-modifying antirheumatic drugs (csDMARD-IR) in phase 2 and 3 RCTs (NCT02066389; NCT02675426) [24, 25]. To assess the comprehensive benefits of upadacitinib, it is important to understand its impact on patient-centric outcomes. To this end, we examined the effect of two doses (15 mg or 30 mg daily) of upadacitinib versus placebo on PROs in SELECT-NEXT, an RCT assessing the efficacy and safety of upadacitinib in moderately to severely active csDMARD-IR RA patients.

## Methods

### Study design and participants

Full details of the study design of SELECT-NEXT (ClinicalTrials.gov, NCT02675426) were reported previously [24]. Patients were randomly assigned (2:2:1:1) to receive either upadacitinib 15 mg or 30 mg or placebo daily for 12 weeks while continuing background csDMARD therapy. After the initial 12-week placebo-controlled period, patients taking placebo received 15 mg or 30 mg of upadacitinib daily, according to the prespecified randomisation assignment. Patients, investigators, and the funder were masked to the treatment allocations. This report is based on post hoc analyses data collected during the placebo-controlled period of SELECT-NEXT. Study participants were ≥ 18 years of age, had active RA for ≥ 3 months, and received csDMARDs for ≥ 3 months with stable doses for ≥ 4 weeks before study entry and inadequate responses to ≥ 1 of the following csDMARDs: methotrexate (MTX), sulfasalazine, or leflunomide. The protocol allowed for enrolment of ≤ 20% with intolerance to at most one biologic DMARD (bDMARD); bDMARD-IR patients were excluded. The protocol was approved by the independent ethics committees or institutional review boards at all study sites. All participants provided written informed consent before enrolment. The RCT was conducted in accordance with the ethical principles that have their origin in the current Declaration of Helsinki and consistent with International Conference on Harmonisation Good Clinical Practice and Good Epidemiology Practices, along with all applicable local regulatory requirements. All patient data were de-identified and complied with patient confidentiality requirements.

### Patient-reported outcomes

PROs were secondary outcome measures in the SELECT-NEXT trial. Patient Global Assessment of Disease Activity (PtGA) and the patient’s assessment of pain were measured using visual analogue scales (VAS) of 0 to 100 mm, with higher scores indicating greater disease activity and worse pain. Reductions of ≥ 10 mm in both PtGA and pain scores are the minimum clinically important difference (MCID). Physical function was assessed by the Health Assessment Questionnaire-Disability Index (HAQ-DI) [26, 27], with higher scores indicating worse physical function and greater disability; a reduction of ≥ 0.22 units is the MCID [28, 29], and a score ≤ 0.25 is the normative value [30]. Fatigue was assessed by the Functional Assessment of Chronic Illness Therapy-Fatigue (FACIT-F) scale; scores range from 0 to 52, with higher scores indicating less fatigue [31], an increase of ≥ 4.0 points is defined as MCID [28], and a score of 43.6 as normative [32]. HRQOL was evaluated using the Medical Outcomes Short Form 36 Health Survey (SF-36), which assesses eight domains (Physical Functioning [PF], Role-Physical [RP], Bodily Pain [BP], General Health [GH], Vitality [VT], Social Functioning [SF], Role-Emotional [RE], and Mental Health [MH]), scored from 0 to 100 and aggregated into the physical component summary (PCS) and mental component summary (MCS) measures [33, 34], with normative values of 50 and standard deviations of 10. The SF-36 domain scores were compared with age- and gender-matched norms. Higher SF-36 scores indicate better health; the MCID is an increase of ≥ 2.5 points for SF-36 PCS and MCS and ≥ 5.0 points for individual SF-36 domains [28, 29]. Euro Qol 5-Dimension 5-Level Questionnaire (EQ-5D-5 L) was also used to assess HRQOL. EQ-5D-5 L has two components: a 0 to 100-mm VAS where 0 represents the worst imaginable health state and 100 represents the best imaginable health state and an index score, which has a maximum score of 1 representing the best health state [35, 36]. AM stiffness severity was reported on a numeric scale of 0 to 10, with higher scores indicating greater severity. Duration of AM joint stiffness was reported by the patient as the length of time, in minutes, that AM joint stiffness lasted on the day before each study visit. Because no values for MCID are reported in the literature, the proposed MCID for AM stiffness severity was defined as a reduction of ≥ 1 point, and the minimal important difference (MID) for AM stiffness duration was proxied at half the standard deviation of the mean baseline values. The Work Instability Scale for RA (RA-WIS) identifies patients at risk for disability-associated work instability, defined as a mismatch between an individual’s functional capabilities and job demands because of RA [37]. RA-WIS scores range from 0 to 23, with higher scores indicating a greater risk of work disability; scores < 10 are considered low risk, and MCID is a reduction of ≥ 5 points [38].

### Statistical analyses

Changes from baseline at weeks 4 and 12, 95% confidence intervals, and nominal *P* values were analysed using a mixed-effect repeated measures model with unstructured variance-covariance matrix including treatment, visit, treatment-by-visit interaction, and prior bDMARD use as fixed factors and baseline value as a covariate. The assumptions of linear regression were checked and met for all outcomes included in the study except for AM stiffness duration and EQ-5D-5 L. Linear regression models were implemented for the analysis of AM stiffness duration and EQ-5D-5 L outcomes for consistency; given the large sample size, estimates are unlikely to be biassed. The results were expressed as least squares mean (LSM) changes. The baseline values and LSM changes for SF-36 domains were transformed based on the mean and standard deviation of the 1998 general US population. Analyses were performed in the full analysis set of all randomly assigned patients who received at least one dose of study drug.

The percentages of patients reporting improvements in PRO scores from baseline to week 12 ≥ MCID or scores ≥ normative values (age- and gender-matched for SF-36 only) at week 12 were compared between active treatment groups and placebo. Non-responder imputation was used when PRO data were missing. Comparisons between active treatment groups and placebo were made using chi-square tests. For each PRO, the incremental numbers needed to treat (NNTs) to achieve clinically meaningful improvements from baseline (≥ MCID or MID) were calculated as the reciprocal of the response rate differences between the active treatment groups and placebo. Times to response from baseline to week 12 were assessed for pain, HAQ-DI, and AM stiffness using Kaplan-Meier analysis. Median times to response were calculated for each dose group; comparisons between the groups used log-rank tests. *P* < 0.05 was considered significant.

## Results

### Study population

A total of 661 patients with active RA were randomised and treated (221 received upadacitinib 15 mg; 219 received upadacitinib 30 mg; 221 received placebo); of these, 618 (93%) completed the placebo-controlled 12-week period (14 patients in the placebo group, 11 patients in the upadacitinib 15-mg group, and 18 patients in the upadacitinib 30-mg group discontinued). Baseline characteristics were balanced across the 3 groups (Table 1). At baseline, 61% of patients had received MTX only, 21% a combination of MTX and another csDMARD, and 19% with only a csDMARD other than MTX. Thirteen percent of patients had prior bDMARD exposure; these patients were either intolerant or had < 3 months exposure to bDMARDs. Patients with an inadequate response to bDMARDs were excluded from entry. Across the groups, Disease Activity Score 28 using C-reactive protein (DAS28[CRP]) ranged from 5.6 to 5.7 and Clinical Disease Activity Index (CDAI) ranged from 37.8 to 38.6 indicating high baseline disease activity in this population.

**Table 1:** Patient demographics and baseline characteristics

Characteristic	PBO, *n* = 221	UPA 15 mg, *n* = 221	UPA 30 mg, *n* = 219
Age (years), mean ± SD	56.0 ± 12.2	55.3 ± 11.5	55.8 ± 11.3
Female, *n* (%)	166 (75.1)	182 (82.4)	172 (78.5)
White, *n* (%)	187 (84.6)	188 (85.1)	186 (84.9)
Duration RA diagnosis (years), mean ± SD	7.2 ± 7.5	7.3 ± 7.9	7.3 ± 7.9
Duration of RA (≥ 5 years), *n* (%)	99 (44.8)	98 (44.3)	102 (46.6)
CDAI, mean ± SD	37.8 ± 11.8	38.3 ± 11.9	38.6 ± 12.7
DAS28-CRP, mean ± SD	5.6 ± 0.8	5.7 ± 1.0	5.7 ± 0.9
Seropositive for RF, *n* (%)	164 (74.2)	163 (73.8)	146 (66.7)
Anti-CCP antibody positive, *n* (%)	167 (75.9)	174 (79.1)	155 (70.8)
Tender joint count (of 68), mean ± SD	24.7 ± 15.0	25.2 ± 13.8	26.2 ± 14.3
Swollen joint count (of 66), mean ± SD	15.4 ± 9.2	16.0 ± 10.0	16.2 ± 10.6
csDMARD use at baseline, *n* (%)			
MTX alone	141 (64.1)	122 (55.5)	136 (62.1)
MTX plus other csDMARD	49 (22.3)	47 (21.4)	39 (17.9)
csDMARD other than MTX	30 (13.6)	51 (23.2)	44 (20.1)
Missing	1 (< 1)	1 (< 1)	0

*CCP* cyclic citrullinated peptide, *CDAI* Clinical Disease Activity Index, *CRP* C-reactive protein, *csDMARD* conventional synthetic disease-modifying antirheumatic drug, *DAS28-CRP* Disease Activity Score 28 using C-reactive protein, *MTX* methotrexate, *PBO* placebo, *RA* rheumatoid arthritis, *RF* rheumatoid factor, *SD* standard deviation, *UPA* upadacitinib

**Fig. 1:**
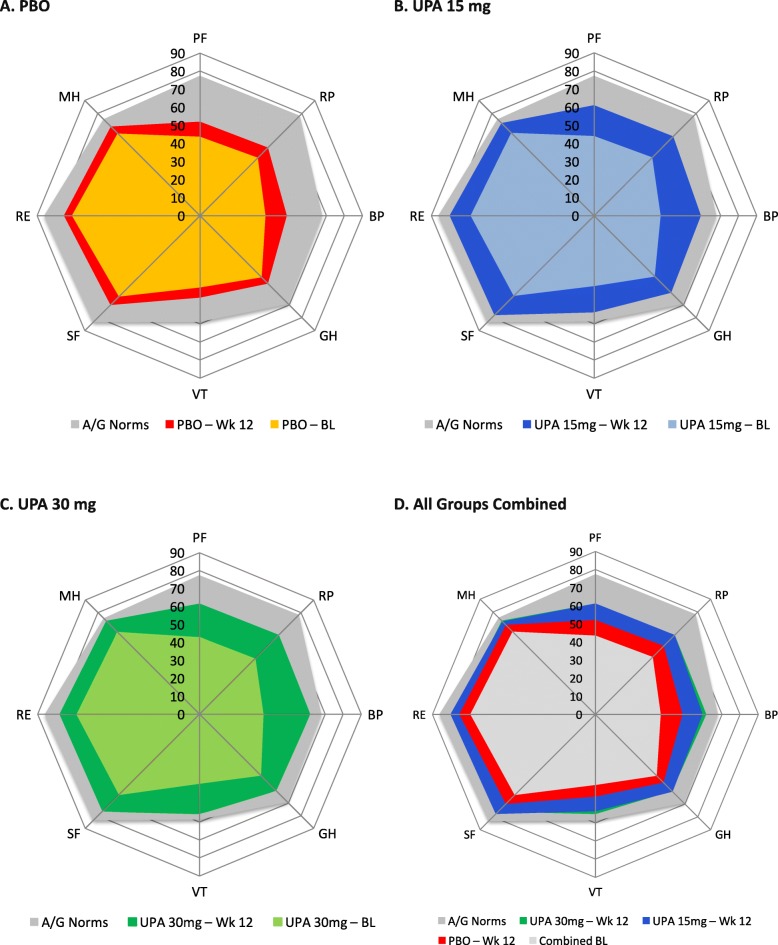
Baseline and post-treatment scores at week 12 across all Short Form 36 Health Survey domains. Baseline (BL) and SF-36 domain scores are relative to age- and gender-adjusted norms (A/G norms) for the general US population. **a** PBO. **b** UPA 15 mg. **c** UPA 30 mg. **d** Combined. In the combined spydergrams, most of the UPA 30-mg results are covered up by the UPA 15-mg results. BL values and SF-36 domain scores were re-scored from 0 to 100. No further transformations were applied for this analysis. BP, Bodily Pain; GH, General Health; MH, Mental Health; PBO, placebo; PF, Physical Functioning; RE, Role-Emotional; RP, Role-Physical; SF, Social Functioning; UPA, upadacitinib; VT, Vitality; Wk, week

Baseline mean PtGA scores ranged from 60.3 to 63.1, mean pain scores from 61.5 to 64.1, mean HAQ-DI scores from 1.4 to 1.5, and FACIT-F from 27.5 to 28.3 across the treatment groups (Table 2). Baseline HRQOL scores (as measured by SF-36 and EQ-5D-5 L) were low. SF-36 PCS was approximately 2.0 standard deviations (SD) < normative values of 50 indicating substantial impairment at baseline (Fig. 1). SF-36 MCS was approximately 0.5 SD less. SF-36 domain scores were low, so that baseline SF-6D utility scores, based on mean scores across all 8 domains [39, 40], were 0.57 in all 3 groups compared with 0.763 in the age/gender-matched normative population. The largest decrements from age and gender norms in both upadacitinib and placebo populations were in physical function (PF, − 33.3 to − 34.7), role physical (RP, − 32.7 to − 34.8), and bodily pain (BP, − 30.9 to − 32.4) domains. Baseline AM stiffness duration ranged from 129 to 152 min and severity from 6.1 to 6.2 (Table 2).

**Table 2:** Baseline PRO scores

PRO measures	Baseline, mean ± SD		
	PBO, *n* = 221	UPA 15 mg, *n* = 221	UPA 30 mg, *n* = 219
PtGA (mm)	60.3 ± 20.5	63.1 ± 21.9	62.8 ± 20.3
Pain VAS (mm)	61.5 ± 20.8	64.1 ± 19.5	64.0 ± 19.8
HAQ-DI	1.4 ± 0.6	1.5 ± 0.6	1.5 ± 0.6
FACIT-F	28.3 ± 11.5	28.1 ± 11.1	27.5 ± 12.6
SF-36 component scores			
PCS	33.1 ± 7.7	33.4 ± 7.4	32.6 ± 7.9
MCS	46.5 ± 11.7	45.9 ± 10.9	46.1 ± 12.0
SF-36 domains^a^			
PF	33.3 ± 9.4	33.5 ± 8.8	32.9 ± 9.7
RP	35.4 ± 8.1	35.5 ± 8.5	34.7 ± 8.9
BP	35.3 ± 6.9	35.5 ± 6.4	34.6 ± 6.8
GH	39.2 ± 9.0	38.9 ± 8.1	39.1 ± 9.4
VT	41.8 ± 9.0	41.5 ± 9.0	41.2 ± 10.0
SF	40.8 ± 10.8	40.6 ± 9.9	40.8 ± 11.0
RE	42.2 ± 11.9	41.1 ± 11.7	41.1 ± 11.9
MH	43.8 ± 10.7	44.1 ± 9.9	44.0 ± 11.5
SF-6D Utility Index	0.57 ± 0.1	0.57 ± 0.1	0.57 ± 0.1
EQ-5D-5 L Index	0.6 ± 0.2	0.6 ± 0.3	0.6 ± 0.3
EQ-5D VAS (mm)	51.4 ± 21.5	49.6 ± 21.3	49.0 ± 22.0
AM joint stiffness			
Duration (min)	138.9 ± 214.0	152.4 ± 241.9	128.6 ± 156.0
Severity^b^	6.1 ± 2.2	6.1 ± 2.4	6.2 ± 2.2
RA-WIS^c^	12.2 ± 6.1	12.9 ± 5.5	13.5 ± 6.5

*AM* morning, *BP* Bodily Pain, *CI* confidence interval, *FACIT-F* Functional Assessment of Chronic Illness Therapy-Fatigue, *GH* General Health, *HAQ-DI* Health Assessment Questionnaire-Disability Index, *LSM* least squares mean, *MCS* mental component summary, *MH* Mental Health, *PBO* placebo, *PCS* physical component summary, *PF* Physical Functioning, *PRO* patient-reported outcome, *PtGA* Patient’s Global Assessment of Disease Activity, *RA-WIS* Work Instability Scale for RA, *RE* Role-Emotional, *RP* Role-Physical, *SD* standard deviation, *SF* Social Functioning, *SF-36* Short Form 36 Health Survey, *UPA* upadacitinib, *VAS* visual analogue scale, *VT* Vitality

^a^The baseline values and LSM changes for SF-36 domains were transformed based on the mean and standard deviation of the 1998 general US population

^b^Assessed on a numeric scale of 1 to 10, with 10 being the worst level

^c^Calculated only for employed patients

### Change from baseline

Statistically significant (*P* < 0.001) LSM changes from baseline to week 12 were reported with both upadacitinib 15 mg and 30 mg compared with placebo for all PROs (*P* < 0.001) except SF-36 MH with 15 mg, and SF-36 MCS, RE, and MH with 30 mg (Table 3). Duration of AM stiffness was reduced 63 to 67% from baseline after initiating upadacitinib.

**Table 3:** LSM change (95% CI) from baseline to weeks 4 and 12

PRO measures	Week 4			Week 12		
	PBO, *n* = 221	UPA 15 mg, *n* = 221	UPA 30 mg, *n* = 219	PBO, *n* = 221	UPA 15 mg, *n* = 221	UPA 30 mg, *n* = 219
PtGA (mm)	− 8.39 (− 11.69, − 5.10)	− 20.22 (− 23.57, − 16.87)***	− 25.06, (− 28.39, − 21.74)***	− 10.36 (− 13.84, − 6.88)	− 29.67 (− 33.18, − 26.16)***	− 30.51 (− 34.03, − 27.00)***
Pain VAS (mm)	− 8.84 (− 12.13, − 5.55)	− 20.86 (− 24.21, − 17.52)***	− 26.33 (− 29.65, − 23.01)***	− 10.26 (− 13.71, − 6.80)	− 29.92 (− 33.40, − 26.44)***	− 31.71 (− 35.20, − 28.22)***
HAQ-DI	− 0.22 (− 0.29, − 0.15)	− 0.43 (− 0.50, − 0.36)***	− 0.44 (− 0.51, − 0.37)***	− 0.26 (− 0.33 to − 0.18)	− 0.61 (− 0.68, − 0.53)***	− 0.55 (− 0.62, − 0.47)***
FACIT-F	2.78 (1.49, 4.06)	5.83 (4.52, 7.14)***	6.24 (4.94, 7.55)***	2.96 (1.62, 4.30)	7.91 (6.56, 9.27)***	7.74 (6.38, 9.11)***
SF-36 component scores						
PCS	2.42 (1.42, 3.42)	5.14 (4.12, 6.16)***	5.91 (4.89, 6.92)***	3.03 (1.88, 4.18)	7.58 (6.43, 8.74)***	8.01 (6.84, 9.18)***
MCS	2.22 (1.02, 3.42)	3.43 (2.21, 4.64)	3.19 (1.98, 4.41)	2.58 (1.30, 3.87)	4.69 (3.40, 5.99)**	3.67 (2.36, 4.98)
SF-36 domains^a^						
PF	2.24 (1.21, 3.26)	4.30 (3.25, 5.34)**	5.00 (3.96, 6.04)***	2.96 (1.80, 4.12)	6.92 (5.75, 8.09)***	6.81* (5.63, 8.00)***
RP	2.30 (1.23, 3.37)	4.17 (3.08, 5.25)**	4.92 (3.84, 6.00)***	2.88 (1.69, 4.08)	6.40 (5.20, 7.61)***	6.36 (5.14, 7.58)***
BP	3.49 (2.42, 4.56)	6.77 (5.68, 7.86)***	8.18 (7.09, 9.26)**	4.66 (3.44, 5.88)	9.44 (8.21, 10.66)***	10.38 (9.14, 11.62)***
GH	1.78 (0.73, 2.82)	4.40 (3.33, 5.46)***	3.35 (2.29, 4.41)*	1.92 (0.78, 3.06)	5.61 (4.47, 6.76)***	5.00 (3.84, 6.15)***
VT	2.53 (1.36, 3.69)	5.14 (3.95, 6.32)***	5.58 (4.40, 6.76)***	2.52 (1.21, 3.83)	6.77 (5.45, 8.09)***	7.05 (5.72, 8.38)***
SF	2.30 (1.05, 3.56)	3.95 (2.67, 5.23)*	3.56 (2.29, 4.83)	2.53 (1.24, 3.82)	6.17 (4.88, 7.47)***	4.81 (3.50, 6.12)**
RE	1.87 (0.60, 3.14)	3.56 (2.27, 4.85)*	3.32 (2.03, 4.61)	2.62 (1.31, 3.94)	5.59 (4.26, 6.91)***	4.10 (2.76, 5.44)
MH	2.67 (1.54, 3.80)	3.61 (2.46, 4.76)	4.13 (2.99, 5.28)*	3.39 (2.14, 4.63)	4.78 (3.53, 6.03)	4.77 (3.51, 6.04)
SF-6D Utility Index	0.04 (0.02, 0.05)	0.06 (0.04, 0.07)*	0.07 (0.06, 0.09)***	0.04 (0.02, 0.05)	0.09 (0.07, 0.10)***	0.09 (0.07, 0.10)***
EQ-5D-5 L Index	0.07 (0.04, 0.10)	0.14 (0.11, 0.17)***	0.14 (0.12, 0.17)***	0.08 (0.05, 0.11)	0.19 (0.16, 0.21)***	0.18 (0.16, 0.21)***
EQ-5D VAS (mm)	4.25 (1.10, 7.39)	9.63 (6.43, 12.83)**	14.09 (10.90, 17.28)***	5.17 (1.82, 8.53)	15.86 (12.48, 19.24)***	17.19 (13.78, 20.61)***
AM joint stiffness						
Duration (min)	− 21.37 (− 40.31, − 2.43)	− 60.73 (− 79.75, − 41.71)**	− 63.06 (− 82.06, − 44.06)**	− 34.27 (− 54.63, − 13.91)	− 85.28 (− 105.61, − 64.95)***	− 85.13 (− 105.65, − 64.62)***
Severity^b^	− 1.03 (− 1.35, − 0.71)	− 2.02 (− 2.35, − 1.70)***	− 2.48 (− 2.80, − 2.15)***	− 1.38 (− 1.73, − 1.03)	− 2.88 (− 3.23, − 2.53)***	− 3.26 (− 3.61, − 2.90)***
RA-WIS^c^	− 1.70 (− 2.78, − 0.62)	− 3.08 (− 4.18, − 1.99)*	− 2.62 (− 3.80, − 1.44)	− 1.55 (− 2.69, − 0.42)	− 4.28 (− 5.41, − 3.14)***	− 3.48 (− 4.72, − 2.24)**

*AM* morning, *BP* Bodily Pain, *CI* confidence interval, *FACIT-F* Functional Assessment of Chronic Illness Therapy-Fatigue, *GH* General Health, *HAQ-DI* Health Assessment Questionnaire-Disability Index, *LSM* least squares mean, *MCS* mental component summary, *MH* Mental Health, *PBO* placebo, *PCS* physical component summary, *PF* Physical Functioning, *PRO* patient-reported outcome, *PtGA* Patient’s Global Assessment of Disease Activity, *RA-WIS* Work Instability Scale for RA, *RE* Role-Emotional, *RP* Role-Physical, *SD* standard deviation, *SF* Social Functioning, *SF-36* Short Form 36 Health Survey, *UPA* upadacitinib, *VAS* visual analogue scale, *VT* Vitality

***P < 0.001 for UPA versus PBO

**P < 0.01 for UPA versus PBO

*P < 0.05 for UPA versus PBO

^a^The baseline values and LSM changes for SF-36 domains were transformed based on the mean and standard deviation of the 1998 general US population

^b^Assessed on a numeric scale of 1 to 10, with 10 being the worst level

^c^Calculated only for employed patients

Statistically significant LSM changes ([95% CI], *P* < 0.001) from baseline were reported as early as week 1 for PtGA (upadacitinib—15 mg, − 10.92 [− 13.87, − 7.97]; 30 mg, − 13.74 [− 16.67, − 10.80] versus placebo, − 3.17 [− 6.07, − 0.27], both *P* < 0.001), pain (upadacitinib—15 mg, − 11.38 [− 14.22, − 8.54]; 30 mg, − 13.80 [− 16.63, − 10.98] versus placebo, − 4.62 [− 7.41, − 1.82], both *P* < 0.001), HAQ-DI (upadacitinib—15 mg, − 0.25 [− 0.31, − 0.19]; 30 mg, − 0.24 [− 0.30, − 0.18] versus placebo, − 0.14 [− 0.19, − 0.08], both *P* < 0.001), AM stiffness severity (upadacitinib—15 mg, − 1.15 [− 1.42, − 0.88]; 30 mg, − 1.25 [− 1.52, − 0.98] versus placebo, − 0.40 [− 0.67, − 0.13], both *P* < 0.001), and AM stiffness duration (upadacitinib—30 mg, − 32.21 [− 46.49, − 17.94] versus placebo, − 9.75 [− 23.96, 4.47], *P* = 0.013).

### Responder analysis

**Fig. 2:**
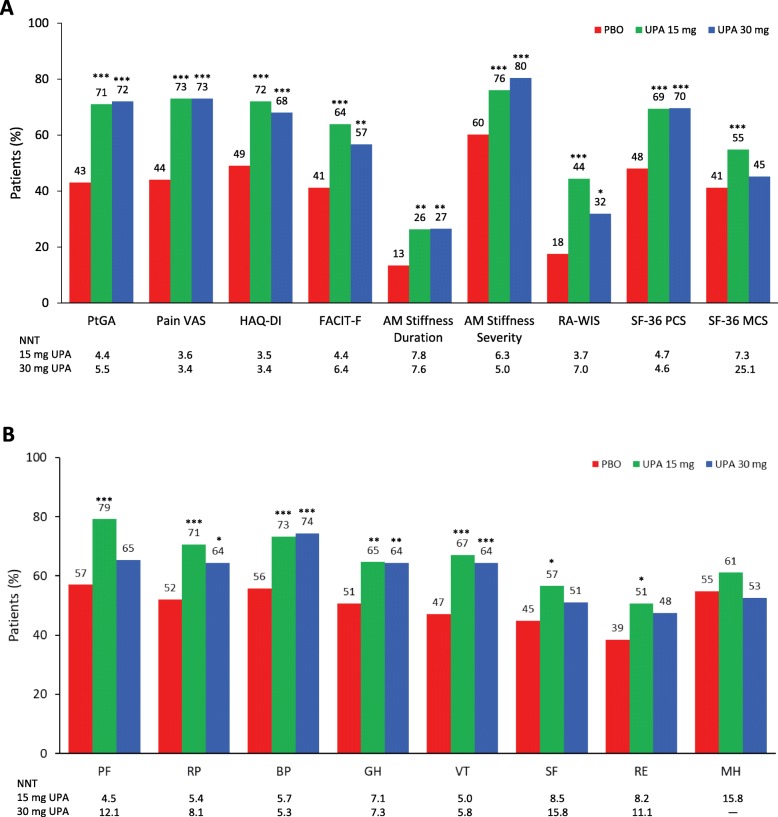
Percentage of patients reporting improvements ≥ MCID at week 12. **a** Patient’s Global Assessment of Disease Activity (PtGA), pain, Health Assessment Questionnaire-Disability Index (HAQ-DI), Functional Assessment of Chronic Illness Therapy-Fatigue (FACIT-F), morning (AM) joint stiffness duration, AM stiffness severity, Work Instability Scale for RA (RA-WIS), and Short Form 36 Health Survey (SF-36). **b** SF-36 individual domains. Baseline values and SF-36 domains were re-scored from 0 to 100. ***P < 0.001, **P < 0.01, *P < 0.5 for upadacitinib versus placebo. BP, Bodily Pain; GH, General Health; MCID, minimum clinically important difference; MCS, mental component summary; MH, Mental Health; NNT, number needed to treat; PBO, placebo; PCS, physical component summary; PF, Physical Functioning; RE, Role-Emotional; RP, Role-Physical; SF, Social Functioning; UPA, upadacitinib; VAS, visual analogue scale; VT, Vitality

At week 12, significantly (*P* < 0.05) more upadacitinib-treated (15 mg and 30 mg) patients reported improvements ≥ MCID in PtGA, pain, HAQ-DI, FACIT-F, duration and severity of AM stiffness, RA-WIS, SF-36 PCS, and SF-36 MCS (15 mg only) and seven of eight SF-36 domains with 15 mg and four of eight SF-36 domains with 30 mg (Fig. 2a, b). Across most PROs, NNTs ranged from four to eight patients; NNTs ≤ 10 are considered clinically meaningful [41].

Patients treated with either dose of upadacitinib had a median time to response of 1 week for pain compared with 4 weeks for placebo; time to response for HAQ-DI and AM stiffness severity was also shorter for upadacitinib-treated patients (1 week for both upadacitinib doses versus 2 weeks for placebo).

**Fig. 3:**
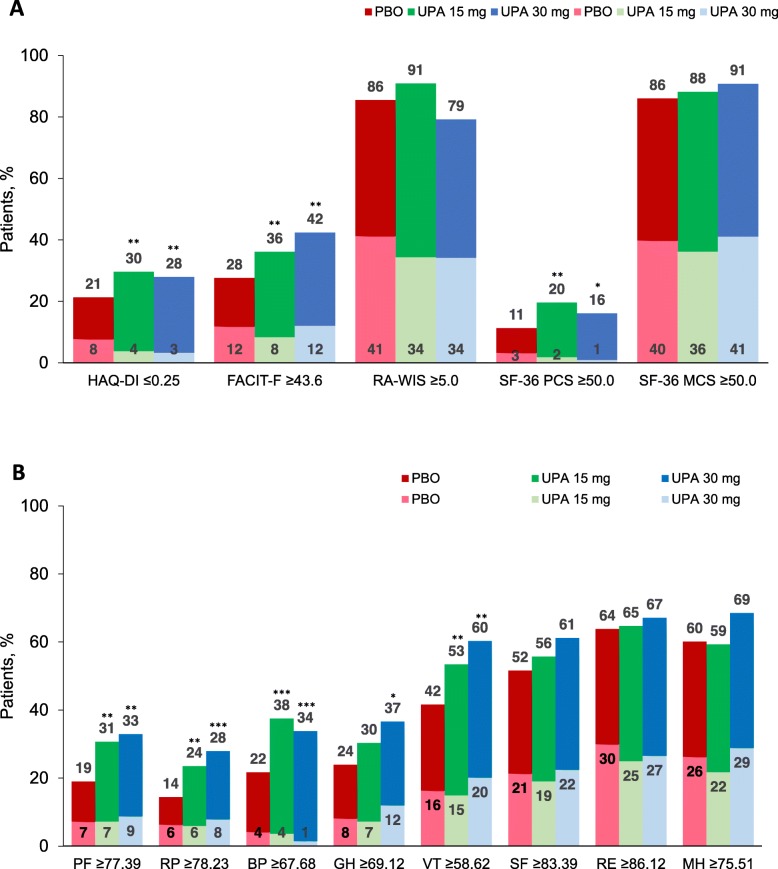
Patients reporting scores ≥ normative values at baseline and week 12. **a** Health Assessment Questionnaire-Disability Index (HAQ-DI), Functional Assessment of Chronic Illness Therapy-Fatigue (FACIT-F), Work Instability Scale for RA (RA-WIS), and Short Form 36 Health Survey (SF-36) PCS and MCS. **b** SF-36 individual domains. Baseline values and SF-36 domains were re-scored from 0 to 100. ***P < 0.001, **P < 0.01, *P < 0.5 for upadacitinib versus placebo. BL, baseline; BP, Bodily Pain; GH, General Health; MCS, mental component summary; MH, Mental Health; PBO, placebo; PCS, physical component summary; PF, Physical Functioning; RE, Role-Emotional; RP, Role-Physical; SF, Social Functioning; UPA, upadacitinib; VT, Vitality; Wk, week

Few patients in any group reported PRO scores ≥ normative values at baseline. The number of patients ranged from 2 (1%) for SF-36 PCS in the upadacitinib 30-mg group to 89 (41%) for SF-36 MCS in the upadacitinib 30-mg group (Fig. 3a). At week 12, the percentage of patients reporting scores ≥ normative values ranged from 18% (SF-36 PCS) to 57% (RA-WIS) with upadacitinib 15 mg and 15% (SF-36 PCS) to 50% (SF-36 MCS) with upadacitinib 30 mg, compared with 8% (SF-36 PCS) to 46% (SF-36 MCS) with placebo (Fig. 3a). Differences between the active and placebo treatment groups were statistically significant (*P* < 0.05) in HAQ-DI, FACIT-F, and SF-36 PCS with both upadacitinib doses versus placebo. Across SF-36 domains, the percentages of patients reporting scores ≥ normative values at 12 weeks ranged from 18% (RP) to 40% (RE) with upadacitinib 15 mg and 20% (RP) to 40% (VT, RE, and MH) with upadacitinib 30 mg, compared with 8% (RP) to 34% (RE and MH) with placebo, statistically significant (*P* < 0.05) in PF, RP, BP, and VT for both upadacitinib doses and GH with 30 mg (Fig. 3b).

## Discussion

Upadacitinib treatment resulted in significant and clinically meaningful improvements in patient-reported disease activity, pain, physical function, fatigue, HRQOL, AM stiffness, and work instability in csDMARD-IR patients with RA. Improvements in PtGA, pain, HAQ-DI, and AM stiffness were reported as early as week 1. Patients not only reported improved PtGA, pain, HAQ-DI, AM stiffness, and FACIT scores, but also reported improvement in SF-36 domain scores that support these outcomes (PF, RP, BP, GH, and VT). There appears to be little difference in the treatment responses between the upadacitinib 15-mg and 30-mg doses, consistent with the reported primary efficacy results [24]. Most PROs assessed resulted in NNTs ≤ 10, which are generally considered favourable [41] and demonstrate the value of upadacitinib treatment for csDMARD-IR patients with RA.

Assessing the effect of upadacitinib on pain, physical function, fatigue, and AM stiffness is important because these outcomes directly impact HRQOL by reducing patients’ ability to perform daily activities and providing barriers to maintaining employment [42–44]. The Work Productivity and Activity Impairment Questionnaire (WPAI) is often used to assess work productivity [45]; however, this measure mainly assesses the time missed from work and impairment while working. Assessing work instability may be a more meaningful measure as it provides a means of screening for possible work disability and an opportunity for individuals to engage in early job retention interventions. In our study, we examined the effect of upadacitinib on work instability using RA-WIS, which identifies patients at risk of work absence or job transitions because of RA [37]. Job transitions are ways in which patients adapt to remain employed and include reducing work hours, taking a short leave of absence, or changing jobs or occupations [10]. Upadacitinib treatment markedly reduced the proportion of patients at risk of work instability. Fatigue is difficult to treat [46, 47], and there is strong evidence for an association between fatigue and other outcomes important from the patient perspective, such as pain, physical function, and depression [6, 43, 48]. This post hoc analysis demonstrated clinically meaningful improvements in fatigue as well as pain and physical function with upadacitinib treatment in csDMARD-IR patients with RA.

This RCT has several strengths of note. Several validated PROs reflecting different aspects of the patient experience were assessed in this study. The analyses performed in this study were comprehensive in nature as they not only examined the changes from baseline but also the proportion of patients reporting improvements ≥ MCID/MID criteria and population norms as well as the time to response for important patient-centric outcomes, such as pain and physical function. The use of MCID or MID criteria to measure response provides a context of how clinically meaningful these improvements are from a patient’s perspective. In addition, assessing the proportion of patients with improvements that reach normative values is a more stringent assessment criterion than MCID, and our results show that a statistically significant proportion of csDMARD-IR patients with RA reported this level of improvement with upadacitinib treatment.

This RCT also has limitations to be considered when interpreting the results. PROs were collected at fixed visits; therefore, responses were unavailable at other time points; however, differences in the outcomes occurred early and were maintained at week 12. The generalisability of these results to patients with milder disease may be limited because patients had moderately to severely active disease at enrolment. The method used to impute missing data (non-response imputation) assumes that missing PRO scores are associated with non-responses, which are stringent conditions and may underestimate the true rate of response.

## Conclusions

Upadacitinib 15 mg and 30 mg daily resulted in rapid and clinically meaningful improvements in the outcomes important to patients including disease activity (per PtGA), pain, physical function, fatigue, HRQOL, AM stiffness, and work instability among csDMARD-IR patients with RA.

## Data Availability

The datasets used and/or analysed during the current study are available from the corresponding author on reasonable request.

## Abbreviations

AM: Morning
bDMARD: Biologic disease-modifying antirheumatic drug
BP: Bodily pain
CI: Confidence interval
csDMARD: Conventional synthetic disease-modifying antirheumatic drug
CCP: Cyclic citrullinated peptide
CDAI: Clinical Disease Activity Index
CRP: C-reactive protein
DAS28-CRP: Disease Activity Score 28 using C reactive protein
EQ-5D-5L: Euro Qol 5-Dimension 5-Level Questionnaire
FACIT-F: Functional Assessment of Chronic Illness Therapy-Fatigue
GH: General Health
HAQ-DI: Health Assessment Questionnaire-Disability Index
HRQOL: Health-related quality of life
IR: Inadequate response
JAK: Janus kinase
LSM: Least squares mean
MCID: Minimum clinically important difference
MCS: Mental component summary
MH: Mental Health
MID: Minimal important difference
MTX: Methotrexate
NNT: Number needed to treat
PBO: Placebo
PCS: Physical component summary
PF: Physical Functioning
PRO: Patient-reported outcome
PtGA: Patient Global Assessment of Disease Activity
RA: Rheumatoid arthritis
RA-WIS: Work Instability Scale for RA
RCT: Randomised controlled trial
RE: Role-Emotional
RF: Rheumatoid factor
RP: Role-Physical
SD: Standard deviation
SF: Social functioning
SF-36: Short Form 36 Health Survey
tsDMARD: Targeted synthetic biologic disease-modifying antirheumatic drug
UPA: Upadacitinib
VAS: Visual analogue scale
VT: Vitality
WPAI: Work Productivity and Activity Impairment Questionnaire
