# Correction to: Upadacitinib improves patient-reported outcomes in patients with rheumatoid arthritis and inadequate response to conventional synthetic disease-modifying antirheumatic drugs: results from SELECT-NEXT

**DOI:** 10.1186/s13075-020-02238-4

**Published:** 2020-06-09

**Authors:** Vibeke Strand, Janet Pope, Namita Tundia, Alan Friedman, Heidi S. Camp, Aileen Pangan, Arijit Ganguli, Mahesh Fuldeore, Debbie Goldschmidt, Michael Schiff

**Affiliations:** 1 Stanford University, Palo Alto, CA USA; 2 University of Western Ontario, London, ON Canada; 3 AbbVie Inc., North Chicago, IL USA; 4 Analysis Group, Inc., New York, NY USA; 5 University of Colorado, Denver, CO USA

**Correction to: Arthritis Res Ther (2019)21:272**

**https://doi.org/10.1186/s13075-019-2037-1**

Following publication of the original article [1], the authors reported an error in some of the data values in Fig. 2a. The correct Fig. 2a can be seen below.

**Fig. 2:**
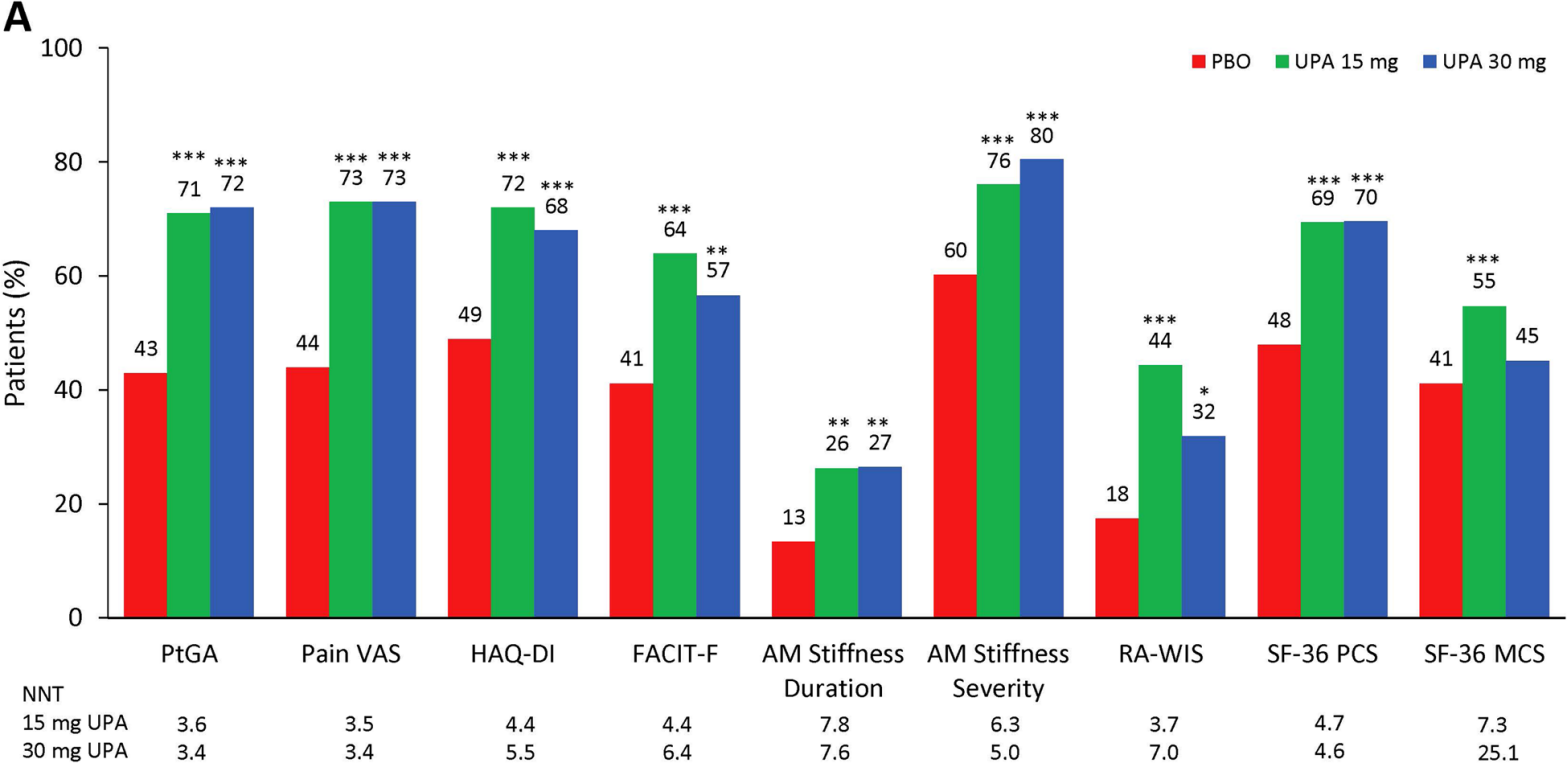
Percentage of patients reporting improvements ≥ MCID at week 12. **a** Patient’s Global Assessment of Disease Activity (PtGA), pain, Health Assessment Questionnaire-Disability Index (HAQ-DI), Functional Assessment of Chronic Illness Therapy-Fatigue (FACIT-F), morning (AM) joint stiffness duration, AM stiffness severity, Work Instability Scale for RA (RA-WIS), and Short Form 36 Health Survey (SF-36)
